# CP-AFM Molecular Tunnel Junctions with Alkyl Backbones Anchored Using Alkynyl and Thiol Groups: Microscopically Different Despite Phenomenological Similarity

**DOI:** 10.1021/acs.langmuir.3c03759

**Published:** 2024-02-13

**Authors:** Yuhong Chen, Ioan Bâldea, Yongxin Yu, Zining Liang, Ming-De Li, Elad Koren, Zuoti Xie

**Affiliations:** † Department of Materials Science and Engineering, Technion-Israel Institute of Technology, Haifa 3200003, Israel; ‡ Department of Materials Science and Engineering, Guangdong Provincial Key Laboratory of Materials and Technologies for Energy Conversion (MATEC), Guangdong Technion-Israel Institute of Technology, 241 Daxue Road, Shantou, Guangdong 515063, China; § Theoretical Chemistry, Heidelberg University, Im Neuenheimer Feld 229, D-69120 Heidelberg, Germany; ∥ Department of Chemistry and Key Laboratory for Preparation and Application of Ordered Structural Materials of Guangdong Province, Shantou University, Shantou 515063, China; ⊥ Quantum Science Center of Guangdong-Hong Kong-Macao Greater Bay Area (Guangdong), Shenzhen-Hong Kong International Science and Technology Park, No. 3 Binglang Road, Futian District, Shenzhen, Guangdong 518048, China

## Abstract

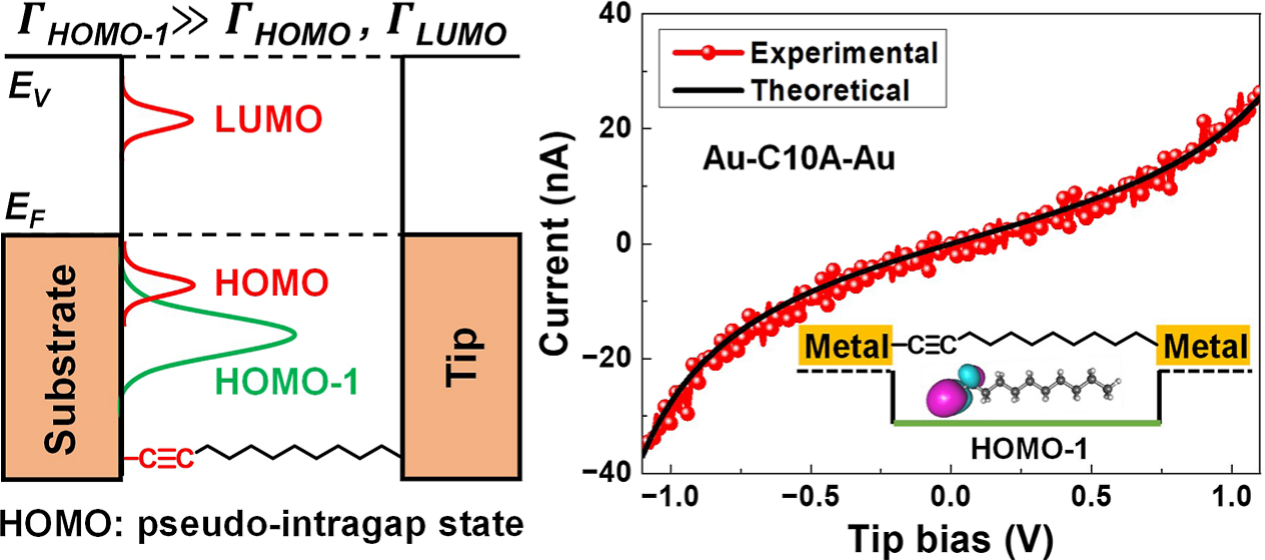

In this paper, we report results on the electronic structure and transport properties of molecular junctions fabricated via conducting probe atomic force microscopy (CP-AFM) using self-assembled monolayers (SAMs) of *n*-alkyl chains anchored with acetylene groups (C*n*A; *n* = 8, 9, 10, and 12) on Ag, Au, and Pt electrodes. We found that the current–voltage (*I*–*V*) characteristics of C*n*A CP-AFM junctions can be very accurately reproduced by the same off-resonant single-level model (orSLM) successfully utilized previously for many other junctions. We demonstrate that important insight into the energy-level alignment can be gained from experimental data of transport (processed via the orSLM) and ultraviolet photoelectron spectroscopy combined with ab initio quantum chemical information based on the many-body outer valence Green’s function method. Measured conductance *G*_Ag_ < *G*_Au_ < *G*_Pt_ is found to follow the same ordering as the metal work function Φ_Au_ < Φ_Au_ < Φ_Pt_, a fact that points toward a transport mediated by an occupied molecular orbital (MO). Still, careful data analysis surprisingly revealed that transport is not dominated by the ubiquitous HOMO but rather by the HOMO–1. This is an important difference from other molecular tunnel junctions with p-type HOMO-mediated conduction investigated in the past, including the alkyl thiols (C*n*T) to which we refer in view of some similarities. Furthermore, unlike in C*n*T and other junctions anchored with thiol groups investigated in the past, the AFM tip causes in C*n*A an additional MO shift, whose independence of size (*n*) rules out significant image charge effects. Along with the prevalence of the HOMO–1 over the HOMO, the impact of the “second” (tip) electrode on the energy level alignment is another important finding that makes the C*n*A and C*n*T junctions different. What ultimately makes C*n*A unique at the microscopic level is a salient difference never reported previously, namely, that C*n*A’s alkyne functional group gives rise to two energetically close (HOMO and HOMO–1) orbitals. This distinguishes the present C*n*A from the C*n*T, whose HOMO stemming from its thiol group is well separated energetically from the other MOs.

## Introduction

Since several decades, molecular electronics has been an active focus of research due to its versatility for studying quantum tunneling phenomena at the molecular scale.^1−27^

Whether in single-molecule setups using the mechanically controlled break junction (MC-BJ)^1,28−34^ or scanning tunneling microscope break junction (STM-BJ)^2,4,5,35−37^ techniques or platforms based on self-assembled monolayers (SAMs) utilized to fabricate “ensemble” molecular junctions containing several dozen molecules via conducting probe atomic force microscopy (CP-AFM)^38−44^ or large area eutectic gallium indium alloy (EGaIn),^45−49^ anchoring groups are indispensable.^50−62^ Along with the ubiquitous thiol,^4,24,36,38,43,44,63−68^ anchoring groups utilized include, e.g., isocyanide,^62,69−71^ nitro,^72−74^ amino,^75−78^ carboxyl,^58,75,79−81^ pyridyl,^80,82,83^ and diazonium.^84−87^

A handful of studies has drawn attention to the fact that alkynes can also be used as the anchoring group to contact molecules on metals of gold^56,57,61,88−90^ and silver.^57,90,91^ SAMs stabilized with alkynes on gold and silver electrodes^56,57,88−91^ have been used to fabricate large-area molecular tunnel junctions with EGaIn top electrode.^57,90^ In addition, single-molecule tunnel junctions with SAMs anchored with alkynes have also been fabricated via STM-BJ^91^ and MC-BJ^61,92^ techniques.

**Figure 1:**
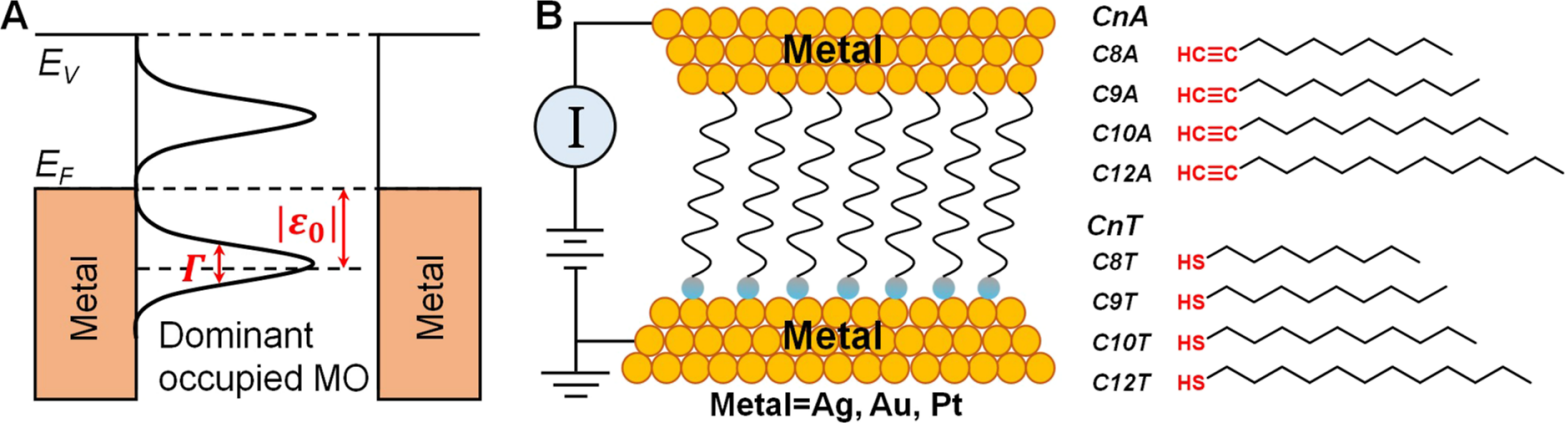
(A) Electronic structure of a molecular junction. (B) Scheme of the CP-AFM molecular junction. A metal-coated (Ag, Au, and Pt) AFM tip is brought into contact with a SAM of alkynes (C*n*A) of various lengths on a metal-coated substrate.

In the present paper, we report experimental and theoretical results for the transport in CP-AFM junctions (Figure 1) fabricated with SAMs of the acetylene-terminated CH_3_–(CH_2_)_*n*−1_–C≡C–H (C*n*A) homologous series (1-decyne, 1-undecyne, 1-dodecyne, and 1-tetradecyne, *n* = 8, 9, 10, and 12, respectively), cf. Figure S1.

Besides, to the best of our knowledge, two additional aspects confer further novelty to the present paper.

(a) Transport data have been reported in the past on large areas of EGaIn junctions fabricated with C*n*A of sizes *n* = 6, 8, 10, and 12 on electrodes of gold^57,90^ and silver.^90^ In addition to the gold and silver electrodes utilized in refs (57 and 90), we also used electrodes made of platinum. Utilizing three electrodes (hence, three distinct values of the work function) enables us to more reliably unravel the impact of the work function on the transport properties.
(b) The current densities *J* reported previously for C*n*A EGaIn junctions at low(er) biases have been analyzed within a phenomenological framework (simplified Simmons model) *J*(*V*) = *J*_0_(*V*) exp(−β*d*).^93^ This approach can provide information on the attenuation constant β and the pre-exponential factor *J*_0_ but does not allow a microscopic description, e.g., in terms of a dominant molecular orbital (MO) characterized by specific energy offsets ε_0_ ≡ *E*_MO_ – *E*_F_ relative to the equilibrium Fermi level (*E*_F_) and interface effective couplings to electrodes Γ (Figure 1A). A quantitative fitting of the *I*–*V* curves in the experimentally accessed bias range (|*V*| < 0.6 V,^90^ |*V*| < 0.5 V^57^) has not been attempted.

In an analysis complementary to the aforementioned studies,^57,90^ we focus here on the microscopic description emerging by combining (i) transport data extracted from full *I*–*V* curves measured up to biases |*V*| = 1.5 V, (ii) ultraviolet photoelectron spectroscopy (UPS) data, and (iii) results of elaborate quantum chemical calculations based on the many-body (beyond DFT)^94^ outer valence Green’s function (OVGF).^95,96^

In our analysis of the transport data, we utilize the compact off-resonant single-level model (orSLM).^97^ Similar to our previous works on CP-AFM junctions anchored with thiol groups,^43,44,66^ we show that this model also accurately reproduces the full *I*–*V* curves measured for the present C*n*A junctions. This enables us to compute the energy offset relative to the Fermi level |ε_0_^trans^| of the MO that dominates the charge transport, which is the key quantity of this paper, wherein the energy-level alignment represents the main focus.

Similar to the case of alkyl-based (C*n*T) and oligophenylene-based (OPT*n*) junctions contacted with thiol,^43,44^ we found that C*n*A junctions with Ag electrodes are less conducting than those with Au electrodes, which are, in turn, less conducting than those with Pt electrodes (conductances *G*_Ag_ < *G*_Au_ < *G*_Pt_). Corroborated with the work function (Φ) ordering Φ_Au_ < Φ_Au_ < Φ_Pt_, this points toward a transport mediated by an occupied MO (ε_0_^trans^ ≡ ε_0_ = −|ε_0_| < 0). The naive intuition would be immediately inclined to assign it as the highest occupied MO (HOMO). This conclusion was indeed validated by our extensive studies on C*n*T and OPT*n*^42−44^ on the basis of the three aforementioned pieces of information.

By corroborating the three aforementioned pieces of information for the present C*n*A, we had a double surprise. First, we found that the MO that dominates the transport in C*n*A is not HOMO but rather the HOMO–1. Second, we found that contrary to the thiol-based CP-AFM junctions studied earlier,^43,44,98^ the “second” (tip) electrode brings about an additional MO energy shift.

These are just the two new findings that make the qualitative difference between the present C*n*A-based junctions and the C*n*T-based junctions, whose transport properties appeared to irrelevantly differ from each other when compared at the phenomenological level of refs (57 and 90). When comparing C*n*A and C*n*T junctions below, we use results similar to our previous results for the latter.^66^

## Experimental Section

Although many experimental details related to this study are similar to our previous studies on CP-AFM junctions (e.g., refs (44 and 66)), they are presented briefly below for the reader’s convenience.

### Materials

Gold nuggets (99.999% pure), silver pellets (99.99% pure), platinum target (99.99% pure), titanium (99.99% pure) evaporation boats, and chromium evaporation rods were purchased from Kurt J. Lesker Co. Contact mode atomic force microscope (AFM) tips (DNP-10 silicon nitride probes) were purchased from Bruker AFM Probes. 1-decyne (C8A, 98%), 1-undecyne (C9A, 98%), 1-dodecyne (C10A, 98%), and 1-tetradecyne (C12A, 97%) used in this study were purchased from Sigma-Aldrich Company.

### Conducting Tip and Sample Preparation

#### Preparing Conductive AFM Tips

Contact-mode AFM tips were coated with either Au or Ag by using a thermal evaporator. The thermal evaporator was housed in a glovebox filled with N_2_ to ensure low levels of H_2_O and O_2_, both less than 0.1 ppm. A 500 Å thin film of gold or silver was deposited on top of a 50 Å Cr adhesion layer. The deposition rate ranged from 0.5 to 1.0 Å per second. After deposition, the coated tips were immediately transferred, without exposure to air, to another glovebox containing the CP-AFM setup for the current measurements. Pt films were prepared by sputtering. A 200 Å thick Pt film was deposited on a 50 Å Ti adhesion layer and immediately transferred to the measurement glovebox.

#### Preparation of Flat Metal Substrates

Template-stripped flat metal substrates were used to grow high-quality SAMs. For flat Ag or Au substrates, a 5000 Å thick layer of Ag or Au was deposited onto clean Si wafers by using a thermal evaporator. Next, silicon chips (1 cm^2^) were attached to the metal surface by gluing them using epoxy (EPOTEK 377, Epoxy Technologies, MA). The epoxy layer was cured by placing the wafers in an oven at 120 °C for 1.5 h. For flat Pt substrates, a 3000 Å thick layer of Pt was sputter-coated onto a clean silicon wafer at a rate of approximately 3 Å per second. On top of the Pt film, a subsequent deposition of 300 Å of Cr and 2000 Å of Au was carried out using a sputtering system. The addition of the Cr/Au film improved the adhesion between the flat Pt substrates and the cured epoxy layer, thereby enhancing the yield. The remaining steps for flat Pt substrates were the same as those for flat Ag and Au substrates. SAMs were formed by immersing template-stripped flat metal substrates into 1 mM ethanol and alkyne solutions of the molecules individually for 20 h. After being rinsed with sufficient ethanol and dried with nitrogen flow, the samples were ready for measurements.

### XPS and UPS Measurements

The X-ray photoelectron spectroscopy (XPS) measurements (Figures S2 and S4) were performed on a PHI Versa Probe III XPS system (ULVAC-PHI) using a monochromated Al *K*_α_ X-ray source (1486.6 eV). The base pressure was 5.0 × 10^–8^ Pa. During data collection, the pressure was ca. 1.0 × 10^–6^ Pa.

The sample was mounted on a piece of double-sided adhesive tape on a sample holder. The X-ray spot size was 200 μm, and the power was 50 W under 15 kV. The XPS of C 1s, S 2p, Ag 3d, Au 4f, and Pt 4f core-level spectra was collected using 55 eV pass energy, 0.125 eV/step, and 20 s per step.

The UPS measurements were performed in the same system as for XPS and using a He I light source (21.2 eV). During data collection, the pressure was lower than 1.0 × 10^–5^ Pa. The UPS spectra were collected using 1.3 eV pass energy, 0.05 eV/step, and 20 s per step, with the takeoff angle set to 45°. The HOMO-Fermi level offsets of C*n*A SAMs ε_0_^UPS^ = −|ε_0_^UPS^| on the metal substrates were measured using UPS. In UPS acquisition, a voltage of −5 V was applied to the sample to obtain the secondary electron cutoff.

### Transport Measurements

The prepared flat metal substrates with SAMs were securely mounted in an AFM. The metal-coated AFM tip, which had been previously prepared with a Au or Ag coating, was brought into contact with the SAM on the substrate surface. This contact was achieved by applying a compressive load of approximately 1 nN. The voltage was applied to the AFM tip by using a Keithley 2635B source meter operated in “DC mode”. The voltage was swept across the tip, while the sample remained grounded. The current–voltage (*I*–*V*) characteristics were recorded during the voltage sweep. All measured *I*–*V* curves were linear at low biases and nonlinear at higher biases. Voltage sweeps to ± (1–1.5) V were applied to extract transition voltages (*V*_*t*±_) from the *I*–*V* curves.

## Theoretical Section

### Off-Resonant Single-Level Model for Tunneling Transport

Within the orSLM of transport utilized to process the measured *I*–*V* curves, the dependence of the tunneling current on bias is expressed as follows^97^
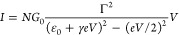
1where *G*_0_ = 2*e*^2^/*h* = 77.48 μS is the conductance quantum, *N* represents the number of molecules per CP-AFM junction, ε_0_ = *E*_MO_ – *E*_F_ is the MO energy offset relative to the equilibrium Fermi level, Γ defined by
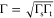
2is the geometrical average of the MO couplings to the substrate (Γ_s_) and tip (Γ_t_), respectively, and γ is a dimensionless quantity characterizing the bias-induced MO shift.

**Figure 2:**
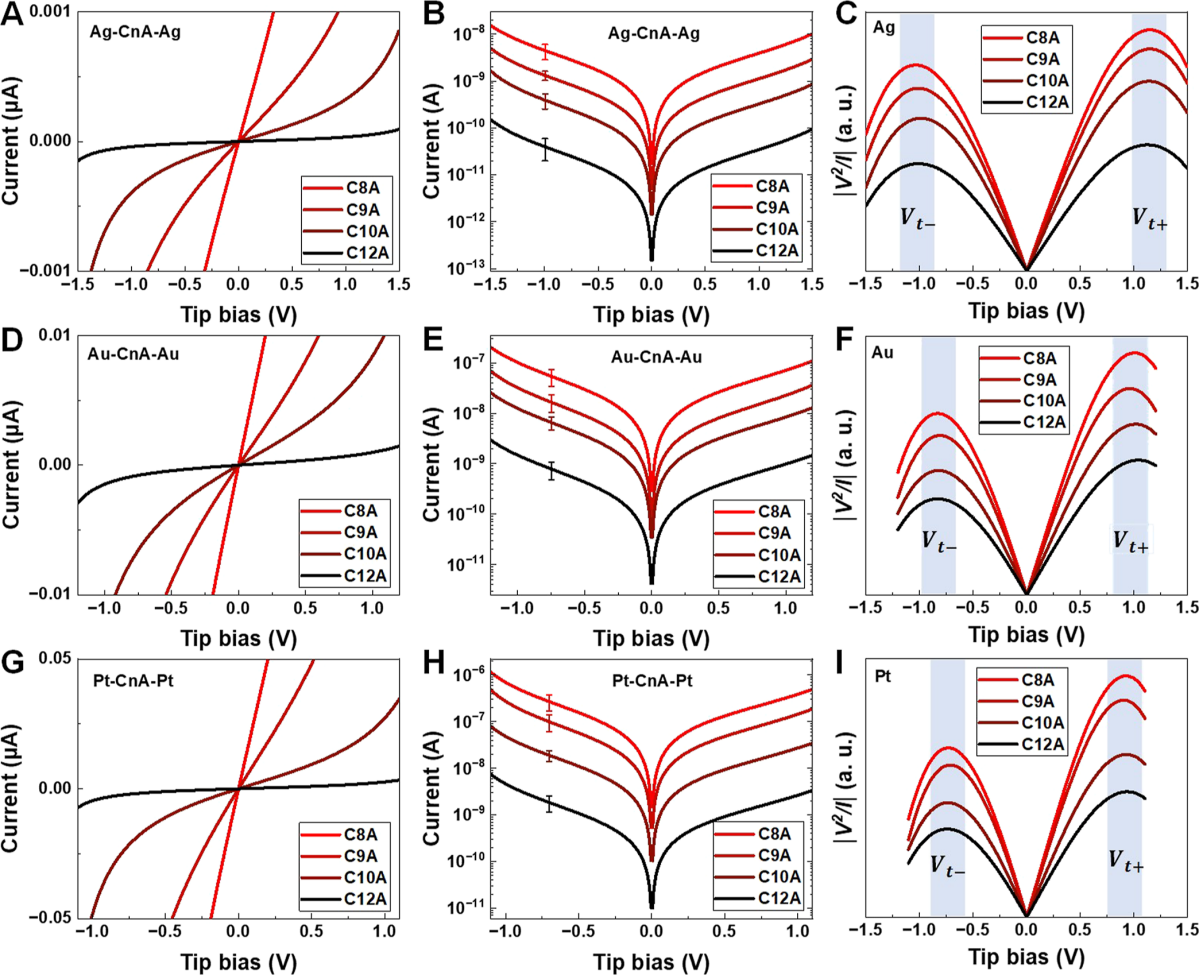
Representative linear and semilog plots of average *I*–*V* curves for (A,B) Ag–C*n*A–Ag, (C,D) Au–C*n*A–Au, and (E,F) Pt–C*n*A–Pt junctions (*n* = 8, 9, 10, and 12).

The MO energy offset ε_0_, which is the main model parameter of interest in the present study, can be obtained by fitting the measured data to eq 1. Alternatively, ε_0_ can be estimated from the transition voltages *V*_*t*+_ and *V*_*t*–_(65) defined as the positive and negative bias at the maximum of *V*^2^/|*I*|^99^ (cf. last column of Figure 2), using the formula^97^
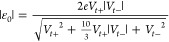
3

In order to avoid possible confusion related to the notation utilized below, we must anticipate a result emerging from the analysis of the C*n*A data (cf. Section “Quantum Chemical Calculations Help Settling a Dilemma: Transport Mediated by HOMO or by HOMO–1?”).

As elaborated in the section “Quantum Chemical Calculations Help Settling a Dilemma: Transport Mediated by HOMO or by HOMO–1?”, the transport in C*n*A junctions is mediated by the HOMO–1 (and not by the HOMO, as in usual cases of junctions with p-type conduction^24,41−44,99^). In cases where a more complete notation is needed to avoid confusion, we will denote the MO offset of the molecules emb(edded) in junctions ε_0_ ≡ *E*_HOMO–1_^emb^ – *E*_F_ < 0 entering eq 1 using the superscripts “trans”, “emb”, or “C*n*A, emb” and write ε_0_^trans^, ε_0_^emb^, or ε_0_^C*n*A,emb^.

On the other side, the quantity determined by our UPS protocol (cf. Figure 7) is the HOMO energy offset of molecules of half junctions (superscript “SAM”, i.e., molecules in SAMs without AFM tips): ε_0_^UPS^ ≡ ε_0_^SAM^ ≡ *E*_HOMO_^SAM^ – *E*_F_ < 0. In the C*n*T junctions, which we will also briefly consider below (cf. Table S2), both ε_0_^trans^ < 0 and ε_0_^UPS^ < 0 refer to the same (HOMO), although they refer to full and half junctions, respectively. For this reason, for C*n*T junctions, we used the positive quantities ε_h_^trans^ = −ε_0_^trans^ and ε_h_^UPS^ = −ε_0_^UPS^.^44^

### Quantum Chemical Calculations

Quantum chemical calculations at the density functional level of theory were carried out to determine the optimized geometries of the isolated C*n*A molecules (Figure S1). For consistency with our previous studies,^42−44,66^ we used the B3LYP hybrid exchange–correlation functional^100−103^ and 6-311++G(d,p) Pople basis sets,^104,105^ as implemented in the Gaussian 16, Revision C.01^106^ package on the bw-HPC platform.^107^ To additionally confirm the correctness of the molecular structures determined (checking always that vibrational frequencies were real), we also performed control calculations using Truhlar’s functional M062x.^108,109^ Differences in geometries optimized using B3LYP and M062x were altogether negligible.

For all sizes considered (*n* ≤ 12), the carbon skeleton is planar, and the linear terminal – C≡C–H group forms an almost *n*-independent angle with the alkyl backbone axis, whose calculated value amounts to ≈147°.

Most importantly, the HOMO and HOMO–1 energies needed in our analysis were obtained from ab initio many-body^94^ quantum chemical calculations using the elaborate OVGF method.^95,96^ As we have insisted previously,^110,111^ the Kohn–Sham (KS) orbitals are mathematical objects rather than true physical/chemical MOs^112,113^ and letting alone that it cannot be utilized for the HOMO–1, difference methods (Δ-SCF^114^ and Δ-DFT^111,115^) enabling estimation of HOMO energies are too inaccurate for the present purpose. Deviations of various methods more or less familiar to the molecular electronics community— Hartree–Fock, DFT/B3LYP, second (MP2), third (MP3), and various flavors of fourth order (MP4’s) Møller–Plesset (MP) expansions— from the OVGF values are presented in Table S3 and Figure S8.

Importantly, Table S3 makes it clear why the present investigation of the energy level alignment in the C*n*A junction can be trusted. As is visible there, unlike any other theoretical method considered, the OVGF-based HOMO energies and the experimental values of the ionization energies^116^ virtually coincide.

The figures depicting molecular geometries and MO spatial distributions were generated with GABEDIT 2.5.1.^116^

## Results and Discussion

### Single-Level Model Analysis of C*n*A Junction Transport Characteristics

**Figure 3:**
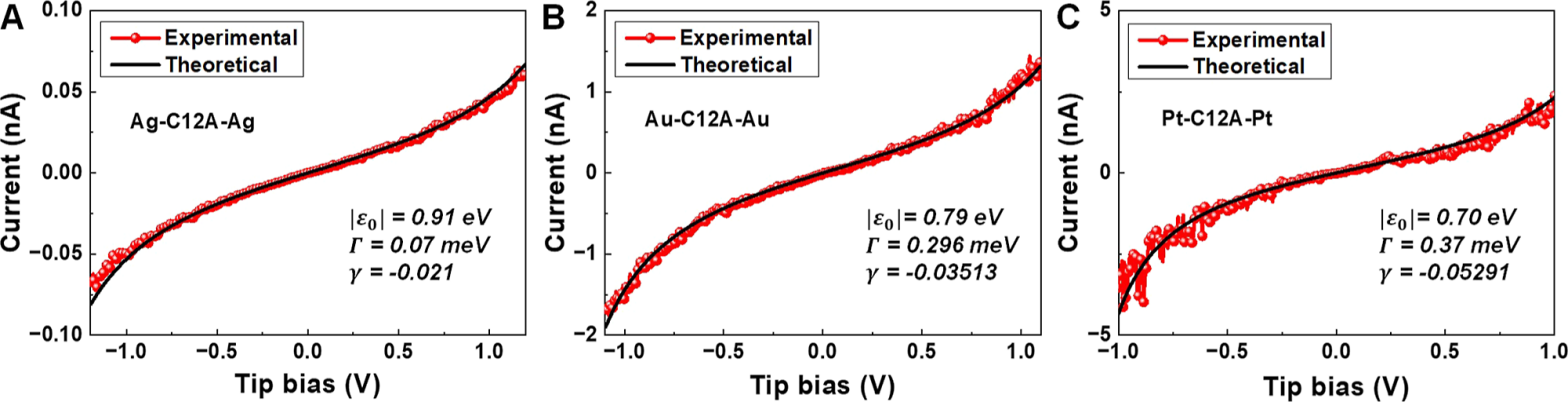
Good agreement between the individual experimental *I*–*V* curves (red) for C12A and those obtained theoretically via eq 1 (black) is illustrated here for (A) Ag/Ag, (B) Au/Au, and (C) Pt/Pt junctions. For each junction, the three parameters entering eq 1—molecule–electrode coupling Γ, MO energy offset |ε_0_| = |ε_0_^trans^|, and γ— are indicated in the legends.

Representative *I*–*V* curves measured for the C*n*A junctions are depicted in Figure 2. Their counterparts measured for C*n*T junctions in conjunction with our earlier study^66^ are shown in Figure S3. Like in previous works on CP-AFM junctions,^24,42−44,67,99,117,118^eq 1 succeeds in reproducing the *I*–*V* curves measured for the present C*n*A junctions. As an illustration in Figure 3, we present curves for C12A junctions and each of the three metal electrodes investigated.

The excellent agreement between the simulated and experimental *I*–*V* curves serves as a robust self-consistency check for the orSLM and confers reliability to the microscopic picture of the tunneling transport in C*n*A junctions emerging from the analysis presented below.

**Figure 4:**
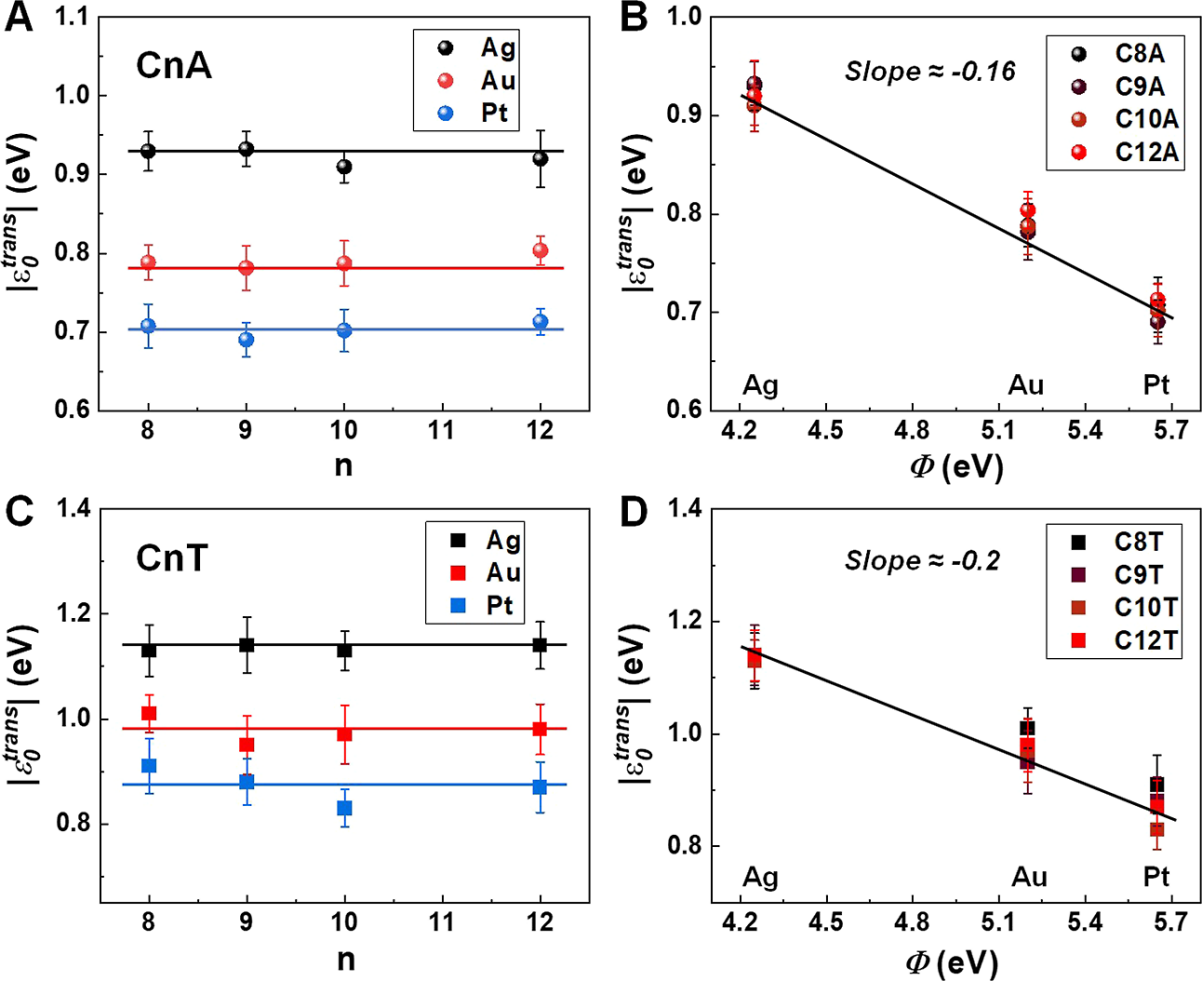
MO energy offset |ε_0_^trans^| of M–C*n*A–M junctions (M = Ag, Au, and Pt) as a function of (A) molecular length and (B) bare electrode work functions. (C,D) Results similar for C*n*T junctions (numerical values from ref (44)). Lines represent linear fits. MO energy offset |ε_0_^trans^| extracted from transport measurements using the orSLM model on C*n*T and C*n*A (*n* = 8, 9, 10, and 12) with Ag, Au, and Pt contacts.

The MO energy offsets |ε_0_^trans^| of the C*n*A junctions extracted from the *I*–*V* characteristics using orSLM are collected in Table 1. As shown in Figure 4A, similar to C*n*T (Figure 4C),^44^ the MO energy offset |ε_0_^trans^| of C*n*A junctions is found to be independent of molecular length (*n*) for each type of metal contact. On the other hand, |ε_0_^trans^| slightly decreases with increasing work function of the contact metals (Figure 4B). This behavior, which is similar to that found earlier for C*n*T junctions (Figure 4D),^44^ demonstrates a strong Fermi level pinning effect in these junctions. In fact, this strong Fermi-level pinning is a common characteristic shared not only with other *n*-alkyl (C*n*T) junctions but also with many other molecular junctions.^24,41−43,66,71,119^

**Table 1:** MO Energy Offsets Deduced from Transport and UPS Measurements for C*n*A CP-AFM Junctions

electrode	quantity	C*n*A			
	*n*	8	9	10	12
	*E*_HOMO_^0^	–9.988	–9.984	–9.981	–9.977
	*E*_HOMO–1_^0^	–10.086	–10.085	–10.084	–10.083
	*E*_HOMO–2_^0^	–10.841	–10.676	–10.543	–10.344
Ag/Ag	|ε_0_^trans^|	0.93	0.93	0.91	0.92
Φ = 4.25 eV	|ε_0_^UPS^|	1.02	0.94	0.96	1.05
Au/Au	|ε_0_^trans^|	0.79	0.78	0.79	0.80
Φ = 5.2 eV	|ε_0_^UPS^|	0.76	0.8	0.76	0.74
Pt/Pt	|ε_0_^trans^|	0.71	0.69	0.70	0.71
Φ = 5.65 eV	|ε_0_^UPS^|	0.74	0.76	0.73	0.72

### Quantum Chemical Calculations Help Settling a Dilemma: Transport Mediated by HOMO or by HOMO–1?

As visible in Figure 2, our C*n*A junctions with Ag electrodes are less conducting than those with Au electrodes, which are, in turn, less conducting than those with Pt electrodes (*G*_Ag_ < *G*_Au_ < *G*_Pt_). Corroborated with the work function (Φ) order Φ_Au_ < Φ_Au_ < Φ_Pt_, this points toward a transport mediated by an occupied MO (ε_0_^trans^ ≡ ε_0_ = −|ε_0_| < 0).

**Figure 5:**
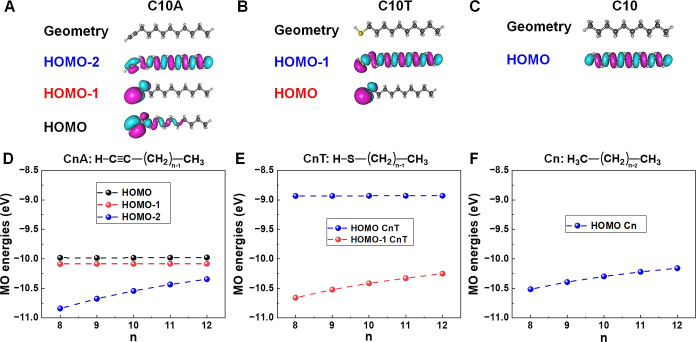
Geometries and relevant MOs of (A) 1-dodecyne (C10A), (B) 1-decanethiol (C10T), and (C) parent decane (C10). MO energies computed via OVGF for species with variable size *N* = 8, 9, 10, and 12 of isolated (D) C*n*A, (E), and (F) C*n* molecules.

For this reason, in our ab initio quantum chemical calculations to isolate C*n*A molecules based on the OVGF method, we focused on the highest occupied orbitals: HOMO, HOMO–1, and HOMO–2. These results are included in Table 1 and depicted in Figure 5D. They reveal that the two highest occupied molecular orbitals (HOMO and HOMO–1) of the C*n*A molecules are close in energy. Their energies are practically independent of molecular size *n*. The next HOMO–2 level is well separated energetically from the HOMO and HOMO–1. Its significant dependence on *n* is a known feature of MOs with spatial extension over the entire chain. It is similar to that of the alkane’s (Cn) HOMO, from which it originates (cf. Figure 5).

In view of the small energy separation between HOMO and HOMO–1 (which cannot be resolved by UPS, see Figure 7), it might be tempting to consider the single level assumed by the (orSLM) approach that succeeded in accurately reproducing the measured currents (cf. Figure 3) to be a combined HOMO–(HOMO–1) contribution (“effective single level”) rather than a genuine single effective level.

**Figure 6:**
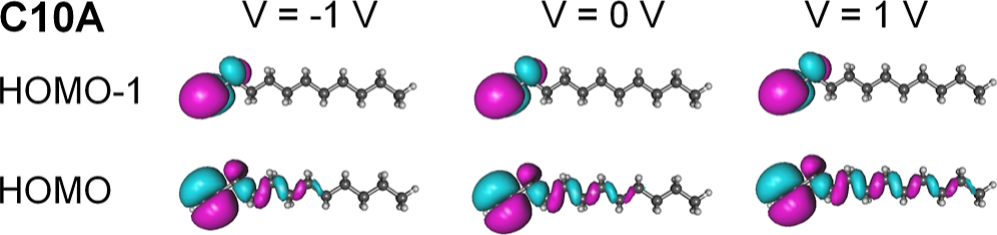
HOMO and HOMO–1 of C10A under bias values indicated in the legend.

Describing the charge transport in the C*n*A junction in terms of an effective (HOMO + HOMO–1) level is a possible working hypothesis. However, for reasons delineated below (see Section S2), we are inclined not to endorse such a scenario. A first useful insight can be gained by comparing the spatial distributions of C*n*A’s and C*n*T’s MOs (Figure 5). Inspection of Figure 5 reveals that it is not the C*n*A’s HOMO but rather the C*n*A’s HOMO–1 of C*n*A that more resembles the C*n*T’s HOMO. Like C*n*T’s HOMO, C*n*A’s HOMO–1 is well localized on the anchoring group, which contrasts to the non-negligible HOMO spatial extension over the C*n*A chain. Combined with the similarity of the transport properties of the C*n*A and C*n*T junctions, a fact already noted by previous studies,^57,90^ we have at least a preliminary hint pointing toward a significant HOMO–1 contribution. Still, this is not the whole issue. The decisive indication that pleads in favor of charge transport dominated by HOMO–1 comes from the behavior of the MOs spatial distribution in the presence of an external field. The effect of an applied bias is depicted in Figure 6. As seen there, biases *V* = ± 1 V comparable to the highest values applied in the experiment (cf. Figures 2 and 3) do significantly affect the HOMO distribution, while that of the HOMO–1 is virtually completely unaffected.

To realize the importance of this behavior, we should return to the good agreement between the measured *I*–*V* curves and the orSLM and emphasize that eq 1 implicitly assumes bias-independent molecule-electrode couplings Γ_s,t_.

To understand why Γ_s,t_ could (hypothetically) depend on bias (a situation never encountered in our previous works based on the orSLM^43,44,67,99^), one should recall the microscopic expression of these quantities^42,97^
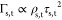
4

The metal’s wide conduction bands legitimately utilize the electrode density of states ρ_s,t_ taken at the Fermi energy in eq 4, which renders Γ_s,t_ quantities independent of energy.^120,121^ However, the molecule-electrode transfer integrals τ_s,t_ can, in general, depend on bias. Loosely speaking, τ_s,t_ represents integrals convoluting molecule-electrode interactions overlapped with products of wave functions describing electrons in electrodes and MOs. Unlike the case of the HOMO–1, whose distribution remains unchanged under bias (compare the HOMO–1 distributions for *V* = −1, 0, and +1 V in Figure 6)
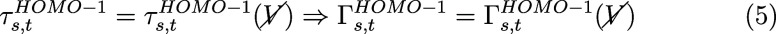
5τ_s,t_ can depend on bias in cases like the HOMO depicted in Figure 6, whose spatial distribution is significantly altered by bias (compare the HOMO distributions for *V* = −1, 0, and +1 V in Figure 6)
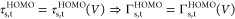
6

To sum up, we rule out a significant HOMO contribution to the transport in C*n*A-based junctions because this would be incompatible with the bias-independent couplings
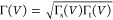
(cf. eq 6), and take the successful data fitting to eq 1— which assumes bias-independent couplings 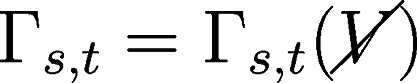 consistent with eq 5— as evidence for conduction mediated by the HOMO–1.

### Does the “Second” Electrode Affect the Level Alignment? Important Insight by Combining UPS, Transport, and Quantum Chemistry Data

The question that we want to address next is: does the “second”(=AFM tip) electrode modify the MO alignment relative to the Fermi level caused by the substrate? This is an important issue because none of the junctions for which this issue was addressed in the past^24,42−44^ were formed with molecules containing a −C≡C– anchoring group.

**Figure 7:**
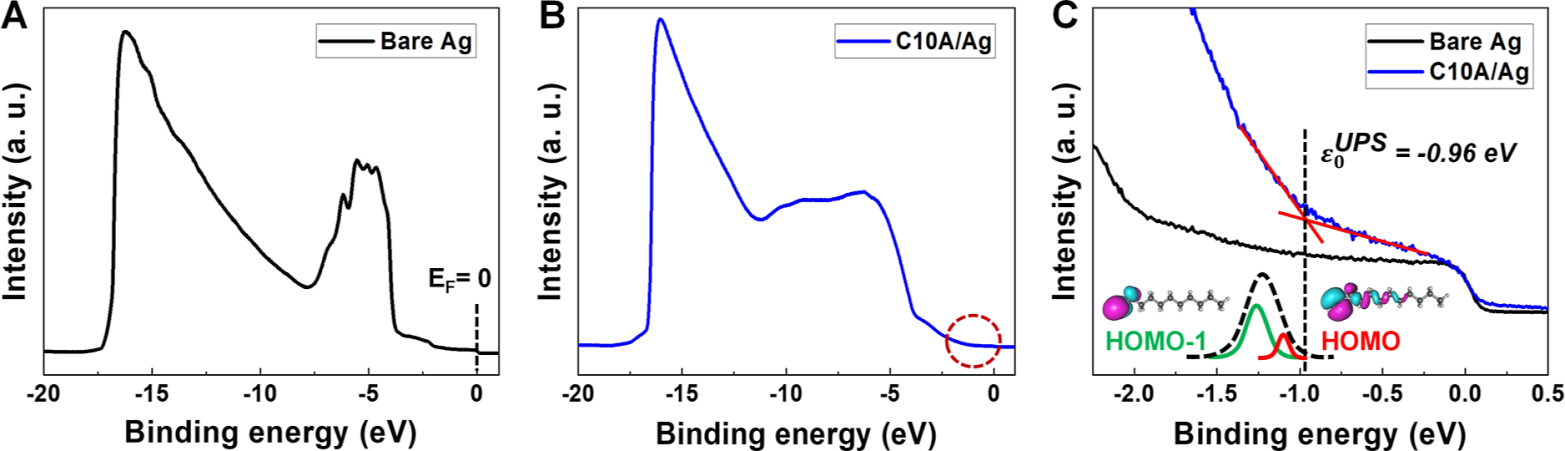
(a) UPS spectra of (a) bare Ag substrate and (b) with adsorbed C10A SAM. (c) Same as (b), but zoomed in at low binding energy, where the HOMO energy of the adsorbed C10A molecule. Because HOMO–1 is energetically very close to HOMO, it cannot be resolved.

UPS is the standard experimental method to investigate occupied electronic states.^122−126^ To obtain additional information about the energy alignment relative to the Fermi level of the substrate, we performed UPS measurements for all C*n*A (*n* = 8, 9, 10, and 12) SAMs on Ag, Au, and Pt substrates. Detailed UPS results are presented in Figures S5 and S6. The standard extrapolation protocol employed earlier^24,41,43,44^ delineated in Figure 7 allowed us to determine the values of the HOMO offset |ε_0_^UPS^| from the UPS data. The values of |ε_0_^UPS^| are also included in Table 1.

Figure 7 makes it clear that, unfortunately, we cannot resolve the energy offset of HOMO–1 from our UPS data. Therefore, in approaching the level alignment problem, we are faced with the following difficulty. The transport data allowed us to compute the energy offset of the HOMO–1 level, but UPS only allows us to determine the HOMO energy offset relative to the substrate electrode; the HOMO–1 offset relative to the substrate cannot be extracted from UPS data.

**Figure 8:**
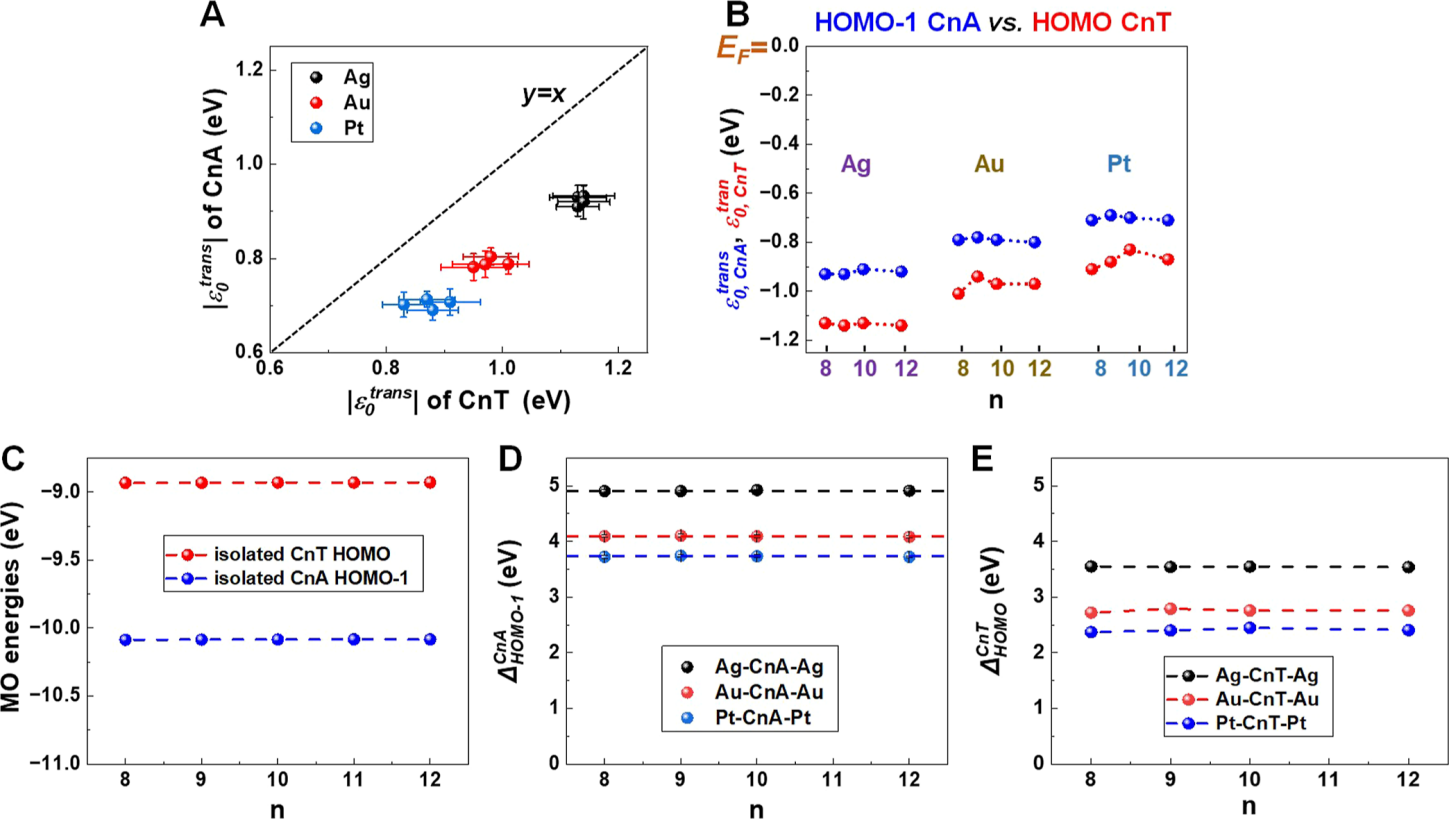
C*n*A and C*n*T properties presented comparatively. (In panels A to C, they are depicted in blue and red color, respectively.) (A,B) MO energy offsets extracted from *I*–*V* curves using the orSLM model. (C) Energies of the pertaining MO energies in isolated C*n*A and C*n*T molecules. (D,E) Differences between MO energies of embedded and isolated molecules computed using eq 10.

The quantum chemical results provide a way out of this difficulty. By means of ab initio OVGF calculations, we can accurately compute the energies *E*_HOMO_^0^ and *E*_HOMO–1_^0^ of the HOMO and HOMO–1 of the isolated C*n*A molecules (cf. Table 1). Although these values pertain to the isolated molecules, their differences are also relevant for molecules embedded in junctions. In estimating the level energies of embedded molecules from those of isolated molecules, applying rigid energy shifts (“scissor” corrections,^127−129^ which do not affect the differences between levels) is common practice:^130^ Applied to our specific case, this means, unlike the absolute HOMO and HOMO–1 energies substantially changing (see, e.g., Figure 8D,E), that the HOMO–(HOMO–1) splitting remains basically unchanged upon molecules’ adsorption or embedding
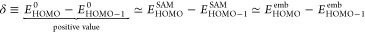
7

Above, subscript 0 refers to isolated molecules.

By using eq 7, we can express the HOMO energy offset of the C*n*A molecules embedded in junctions defined by ε_HOMO_^emb^ ≡ *E*_HOMO_^emb^ – *E*_F_ as follows
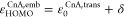
8

**Figure 9:**
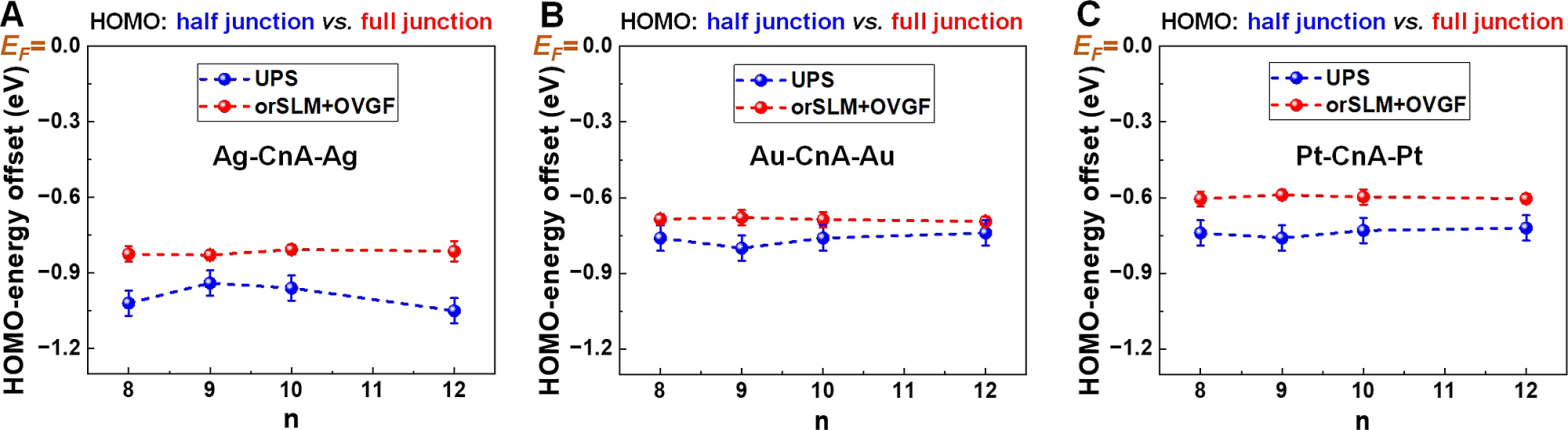
HOMO offset directly measured via UPS clearly differs from the HOMO offset deduced from transport data via orSLM and OVGF quantum chemical calculations.^95,96^ This demonstrates that, contrary to alkane^44^ and oligophenylene^43^ mono- and di-thiols, the (“second”) tip electrode brings about an additional non-negligible (measurable) HOMO shift toward the metal Fermi level. Our data by no means indicate an energy difference monotonically decreasing with the molecular size, a fact that rules out an image charge effect and is consistent to the fact that acetylenes form string covalent bonds with metal electrodes.

The HOMO offset of embedded C*n*A molecules thus derived via the orSLM + OVGF combination can now be compared with the HOMO energy offset ε_HOMO_^C*n*A,SAM^ = ε_0_^UPS^ pertaining to half-junctions directly extracted from the UPS data. These two quantities are depicted in Figure 9 by red and blue symbols, respectively.

The difference between ε_0_^UPS^ and ε_HOMO_^C*n*A,emb^ is positive. It represents the contribution of the “second” (=AFM tip) electrode. The C*n*A molecules in junctions have a HOMO closer to the Fermi level than those in SAMs not contacted to the tip (half-junctions). The values of this difference— which amount up to 0.24 eV and up to 30% of the absolute values—decidedly exceed the standard deviations and are therefore statistically relevant.

Conversely, one can compare the HOMO–1 energy offset (“in junction”) directly extracted from the transport data with the HOMO–1 energy offset (“in half-junction”) by correcting the HOMO energy offset measured via UPS |ε_0_^UPS^| with the ab HOMO–HOMO–1 splitting (eq 7)
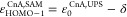
9

The results obtained in this way are depicted in Figure S7, which conveys the same message as those of Figure 9: the additional HOMO–1 shift brought about by the “second” electrode is measurable.

### Comparison between C*n*T and C*n*A Tunnel CP-AFM Junctions

In our earlier studies,^43,44^ detailed results for CP-AFM junctions M–C*n*T–M with M = Ag, Au, and Pt electrodes were reported. Comparison with the present results for C*n*A junctions reveals that C*n*A junctions are only slightly more conductive than C*n*T junctions with the same electrodes and the same number of methyl repeat unit *n*. This behavior is in line with previous work on large-area junctions of Au–C*n*A/EGaIn and Au–C*n*T/EGaIn, wherein their electric properties were found to be indistinguishable or marginally distinguishable (a factor of 2).^57^

To facilitate comparison between the previously investigated C*n*T^44^ and the present C*n*A CF-AFM junctions, we depict relevant aspects in Figures 4, 5, and 8.

As representatives of the C*n*A and C*n*T homologous series, we show in Figure 5, a few orbitals of the ten-member species C10A and C10T along with the parent *n*-decane C10T. As seen there, the C10’s HOMO evolves into the C10T’s HOMO–1 and the C10A’s HOMO–2. Their spatial extension over the molecular chain reflects itself into a similar and significant dependence on the size *n* of the corresponding homologous series depicted by the blue lines in Figure 8A–C.

Interestingly, addition of the terminal thiol group −S–H gives rise to a single occupied MO above the C10’s HOMO (i.e., C10T’s HOMO), while addition of the alkyne group −C≡C–H gives rise to two occupied MOs above the highest occupied orbital of the C10s parent. Noteworthy, it is not the highest but rather the lowest of these two orbitals of C10A (i.e., C10A’s HOMO–1) that is similar to C10T’s HOMO, which is the dominant orbital for conduction in C10T junctions.^44^

Importantly, both in the isolated C*n*A and C*n*T molecules (cf. Figure 8C) and in the embedded C*n*A and C*n*T molecules (cf. Figure 4A,C, respectively), the MO that dominates the transport (HOMO–1 in C*n*A and HOMO in C*n*T) have energies practically independent of *n*.

The similarity of the C*n*A’s HOMO–1 and C*n*T’s HOMO spatial distributions is perhaps the most eye-catching feature visible in Figure 5. It makes intuitively understandable why, upon molecule embedding, the C*n*A’s HOMO–1 located at the molecule’s end should hybridize with the neighboring metal atoms in a manner similar to C*n*T’s HOMO hybridization with the metal substrate. In turn, this indicates that the MO-electrode couplings Γ (the quantity entering eq 1) of C*n*A and C*n*T should be similar.

This point (misplaced in this paper devoted to energy-level alignment) will be discussed elsewhere. However, we still note that our conclusion on the similar coupling to electrodes of the dominant MO in C*n*A and C*n*T, which emerged from the analysis of the microscopically calculated MO spatial distributions, provides a microscopic rationale for a similar conclusion proposed previously on a pure phenomenological basis.^90^ In this vein, the values of the MO energy offsets in C*n*A slightly smaller than those of C*n*T (|ε_0_^C*n*A^| ≃ |ε_0_^C*n*T^|−0.2 eV, see Figure 8A,B) explain the slightly larger values of the conductance *G* of the former junctions (recall that *G* = *NG*_0_Γ^2^/ε_0_^2^ ∝ 1/ε_0_^2^, an expression deduced from eq 1(42,97)).

Noteworthy in the context of energy-level alignment is another fact. While the offset value characterizing the dominant HOMO–1 transport channel in C*n*A is slightly smaller than the dominant HOMO transport channel in C*n*T (cf. Figure 8A,B), the difference between the corresponding energies in isolated C*n*A and C*n*T is much larger and has an opposite sign (cf. Figure 8D,E). The C*n*A’s HOMO–1 lies below the C*n*T’s HOMO: *E*_0,C*n*A_^HOMO–1^ – ε_0,C*n*T_^HOMO^ ≈ −1.2 eV versus ε_0_^C*n*A^ – ε_0_^C*n*T^ ≈ 0.2 eV. The contrast between the energy levels of the isolated and embedded C*n*A and C*n*T molecules becomes evident by comparing, e.g., Figure 8B on one side with Figure 8D,E on the other side.

To compare the location relative to the metal’s Fermi levels of the dominant MOs belonging to the embedded C*n*A and C*n*T molecules (superscript embed) with the location of the MOs belonging to the isolated molecules (superscript 0), one can inspect the following quantities
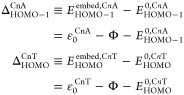
10where Φ is the work function of the bare metal. Traditionally, these differences between MO energies of embedded and isolated molecules are related to the electron density rearrangement that can be seen as partial charge transfer through covalent molecule-electrode bonds, giving rise to interface dipoles.^123^

Just a reminder before moving on to the next section, in our previous work,^44^ we concluded that image charge effects do not play a significant role in C*n*T junctions.

### Why Image Charge Effects Are Not Important

Although image charge effects may play a role in energy level alignment at organic–metal interfaces,^123,131^ we have not included them in the foregoing discussion. The reason is the following: if interaction with image charges was important, the magnitude of the image-driven MO shift would monotonically decrease with increasing molecular size *n*. Then the energy offsets |ε_0_| would monotonically depend on the molecular size *n*, but the data directly extracted from the experiment depicted in Figure 9 does not support this behavior.

In alternative terms, Figure 8D,E convey the same message. If size-dependent MO shifts due to charge images were important, the differences Δ defined by eq 10 between size-dependent energies of embedded molecules and size-independent energies of isolated molecules (cf. Figure 8B and Table 1) would exhibit a significant monotonic dependence on *n*. As seen in Figure 8D,E, this is not the case. The physical/chemical reason should be clear: in steady-state regime, the molecule is not significantly charged during an off-resonant tunneling process.

### Final Remarks

A junction exhibiting an n-type conduction dominated by the LUMO (or another unoccupied MO) would be more conductive if the electrodes had a lower work function. Our measurements for C*n*A junctions have shown that the contrary is true (*G*_Ag_ < *G*_Au_ < *G*_Pt_ and Φ_Ag_ < Φ_Au_ < Φ_Pt_).

Corroborated with the behavior *G*_Ag_ < *G*_Au_ < *G*_Pt_ versus Φ_Ag_ < Φ_Au_ < Φ_Pt_— which points toward a conduction dominated by an occupied orbital— and with the analysis of Section “Quantum Chemical Calculations Help Settling a Dilemma: Transport Mediated by HOMO or by HOMO–1?” indicating the predominance of the HOMO–1 over the HOMO
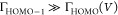
we can intuitively liken the HOMO’s behavior in C*n*A junctions, whose distribution is concentrated at the molecule–substrate interface, with the role of ineffective intragap states ubiquitous at thin film/metal interfaces.

## Conclusions

Previous works on large-area EGaIn junctions^57,90^ have reported that *n*-alkyl with alkyne (C*n*A) and thiol (C*n*T) anchoring groups possess indistinguishable or marginally distinguishable measured currents. Aware of the limitations of their phenomenological transport description (simplified Simmons model), they also stated, e.g., that this “does not imply definitively that there are no differences in the electronic structure or...the shape of the tunneling barrier...”.^57^

Our present paper demonstrated that, definitely, microscopic differences between the C*n*A and C*n*T junction exist. In contrast to the Simmons model, our orSLM^97^ validated and utilized for the presently investigated CP-AFM junctions fabricated with C*n*A, and silver, gold, and platinum electrodes allowed us to extract and microscopically analyze the MO energy offset |ε_0_| (“tunneling energy barrier” in the loose terminology of the Simmons model) from the full *I*–*V* curves measured.

With additional information gathered from UPS data and quantum chemical calculations, we were able to reveal (a) the prevailing role of HOMO–1 in C*n*A molecular junctions as well as (b) the significant role of the “second” (AFM tip) electrode in establishing the energy-level alignment in the C*n*A CP-AFM junctions investigated. None of the two aforementioned findings could have been expected based on naive intuition, given the similarity of the current measured in C*n*A and C*n*T junctions.

Placed in a methodological context, the present paper on CP-AFM junctions of *n*-alkynes is a plea in favor of concurrent transport, UPS, and quantum chemical investigation in gaining insight into the energy-level alignment in tunnel molecular junctions. This is in vein with the methodology adopted in our earlier joint experimental-theoretical works on other molecular tunnel junctions.^42−44,67,118^ Moreover, this is an important step further. We have shown what (due to missing experimental data) we could not show in our precedent studies, namely, that within the experimental errors, the theoretical OVGF-based HOMO values and the experimental ionization energies coincide.

## Supporting Information Available

Molecular geometries, additional transport, XPS, UPS data, and additional results of model and quantum chemical calculations (PDF)
