# Supplementary material for: CP-AFM Molecular Tunnel Junctions with Alkyl Backbones Anchored Using Alkynyl and Thiol Groups: Microscopically Different Despite Phenomenological Similarity
Source: Langmuir. 2024 Feb 13;40(8):4410–23. doi: 10.1021/acs.langmuir.3c03759 (PMC10906003; doi:10.1021/acs.langmuir.3c03759)
Supplement: Supplementary file 1 — la3c03759_si_001.pdf [file la3c03759_si_001.pdf]

Supporting Information — CP-AFM Molecular Tunnel Junctions with Alkyl Backbones Anchored Using Alkynyl and Thiol Groups: Microscopically Different despite Phenomenological Similarity

Yuhong Chen,[†,‡] Ioan Bâldea,[*,¶] Yongxin Yu,[‡] Zining Liang,[‡] Ming-De Li,[§] Elad Koren,[†] and Zuoti Xie[*,†,‡,∥]

†*Department of Materials Science and Engineering, Technion-Israel Institute of Technology, Haifa 3200003, Israel*

‡*Department of Materials Science and Engineering, Guangdong Provincial Key Laboratory of Materials and Technologies for Energy Conversion (MATEC), Guangdong Technion-Israel Institute of Technology, 241 Daxue Road, Shantou, Guangdong, 515063, China*

¶*Theoretical Chemistry, Heidelberg University, Im Neuenheimer Feld 229, D-69120 Heidelberg, Germany*

§*Department of Chemistry and Key Laboratory for Preparation and Application of Ordered Structural Materials of Guangdong Province, Shantou University, Shantou 515063, China*

∥*Quantum Science Center of Guangdong-Hong Kong-Macao Greater Bay Area (Guangdong), Shenzhen-Hong Kong International Science and Technology Park, NO.3 Binglang Road, Futian District, Shenzhen, Guangdong, 518048, China*

E-mail: ioan.baldea@pci.uni-heidelberg.de; zuoti.xie@gtiit.edu.cn

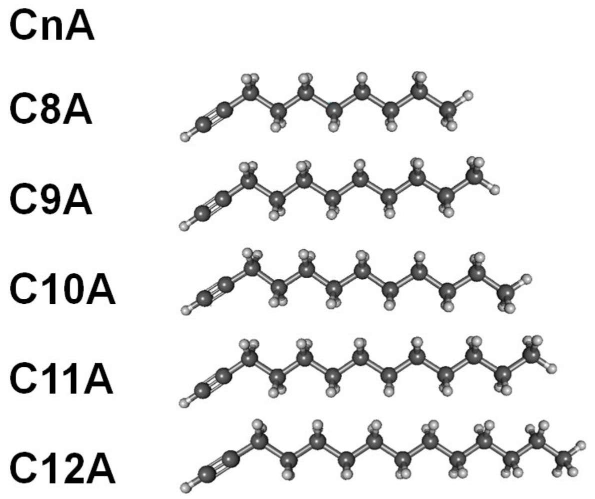

Figure S1: Geometry of CnA molecules optimized at the DFT/B3LYP/6-311++G(d,p) level of theory.

Thickness of CnA Junction determined by XPS measurements

We utilized X-ray photoelectron spectroscopy (XPS) to confirm the presence of a CnA monolayer on metal surfaces and to estimate its thickness. By collecting photoelectrons at specific angles with respect to the surface, we increased the emission path lengths in the film by a factor of sinα, as illustrated in Figure S2A. The attenuation of the XPS signal was expressed as

$$I = I_0 \exp\left(-\frac{d}{\lambda \sin \alpha}\right)$$

where I_0 represents the photoelectron intensity from the bare substrate, I is the intensity from the monolayer-covered samples, d is the thickness of the monolayer, λ is the attenuation length, and α is the take-off angle between the surface and analyzer axis. In our experiment, we collected Ag 3d, Au 4f, and Pt 4f photoelectrons at a take-off angle of 45 degrees. Previous studies have reported attenuation lengths for Ag 3d and Au 4f photoelectrons in organic SAMs of 3.6 nm and 4.2 nm, respectively. Considering that $\lambda \propto \sqrt{E}$, where E is the photoelectron kinetic energy, and that the Pt 4f core level binding energy is only slightly

smaller than the Au 4f core level binding energy, we assumed that the attenuation length for Pt 4f is also close to 4.2 nm.

The SAMs of CnA on Ag, Au, and Pt substrates underwent characterization using X-ray Photoelectron Spectroscopy (XPS). Core level photoelectron signals for C 1s, Ag 3d, Au 4f, and Pt 4f were acquired with a take-off angle of 45°. As illustrated in Figure S4, the intensity of the C 1s signal increased, while the intensities of Ag 3d, Au 4f, and Pt 4f signals decreased as the number of carbon atoms in CnA molecules increased.

Based on this contrast, we calculated the thickness (d) of the SAM for different samples (Table S1, Figure S2A), with the listed values representing the averages of 2—3 samples. Table S1 reveals that the thickness of the CnA SAM increases with the number of CH_2 units.

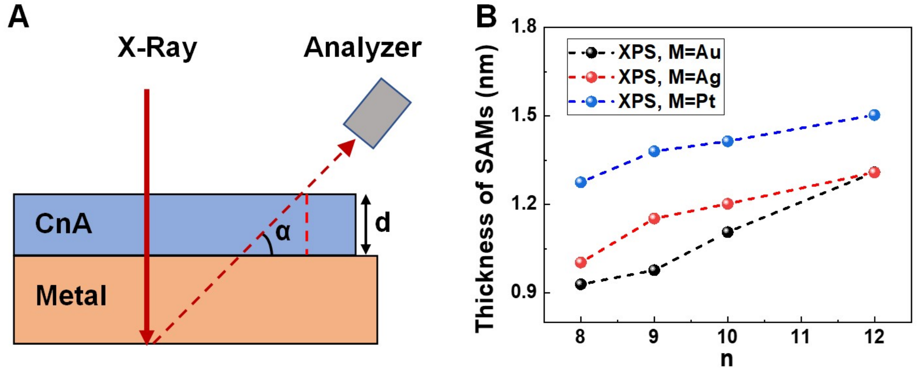

Figure S2: (A) XPS setup for SAM thickness measurements. (B) Dependence of SAM thickness on the molecular size for CnA adsorbed on the metal electrodes investigated in the present work.

Table S1: Thickness in Å of the CnA SAMs on metals obtained via XPS

n	XPS Ag	XPS Au	XPS Pt
8	10.03	9.29	12.75
9	11.52	9.77	13.80
10	12.02	11.06	14.14
12	13.08	13.09	15.03

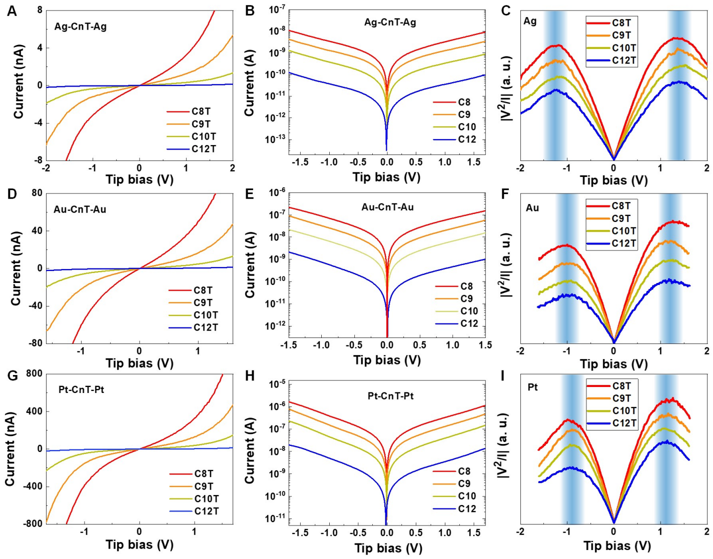

Figure S3: Representative linear and semi-log plots of average I-V curves for (A, B) Ag-CnT-Ag, and (C, D) Au-CnT-Au and (E, F) Pt-CnT-Pt junctions (n=8, 9, 10, 12). Results similar to those previously reported in ref. 66 (Chem. Sci. 2018, **9**, 4456–4467, http://dx.doi.org/10.1039/C8SC00938D).

HOMO and/or HOMO-1 mediated conduction?

In view of the small energy separation between HOMO and HOMO-1 ($E_{HOMO} - E_{HOMO-1} \simeq 0.2\,\mathrm{eV}$, cf. Table 1), it might be tempting to consider the single level assumed by the (orSLM) approach that succeeded in accurately reproducing the measured currents (cf. Figure 3) to be an additive contribution of the HOMO and the HOMO-1 ("effective single level") rather than a genuine single effective level

$$\begin{aligned} I &= G_0 \frac{\Gamma^2_{HOMO}(V)}{\left[E^{emb}_{HOMO}(V) - E_F\right]^2 - (eV/2)^2} V \\ &+ G_0 \frac{\Gamma^2_{HOMO-1}\cancel{(V)}}{\left[E^{emb}_{HOMO-1}(V) - E_F\right]^2 - (eV/2)^2} V \\ &\approx \frac{G_0 V \Gamma^2_{eff}(V?)}{\left[E_{eff}(V) - E_F\right]^2 - (eV/2)^2} \end{aligned}$$

Speaking in general, given the fact that $\Gamma_{HOMO} = \Gamma_{HOMO}(V)$ depends on bias while $\Gamma_{HOMO-1} = \Gamma_{HOMO}\cancel{(V)}$ does not (see discussion related to Figure 6 in the main text),

depending on their interplay Γ_{eff} can or cannot significantly depend on bias. More precisely

$$I \xlongequal[\text{comparable}]{\Gamma_{HOMO}(V) \text{ and } \Gamma_{HOMO-1}} \frac{G_0 V \Gamma^2_{eff}(V)}{\left[E_{eff}(V) - E_F\right]^2 - (eV/2)^2} \tag{S1}$$

$$I \xlongequal{\Gamma_{HOMO}(V) \ll \Gamma_{HOMO-1}} \frac{G_0 V \Gamma^2_{eff}\cancel{(V)}}{\left[E_{eff}(V) - E_F\right]^2 - (eV/2)^2} \tag{S2}$$

Data fitting using eq (1) could not be satisfactory if eq (S1) applied, i.e. if $\Gamma_{eff} = \Gamma_{eff}(V)$ significantly depended on bias, because Γ entering eq (1) is a number that does not depend on V. This implies that eq (1) is precisely like eq (S2), which applies for $\Gamma_{HOMO}(V) \ll \Gamma_{HOMO-1}$. In other words, although slightly closer to the Fermi level, in CnA the HOMO is much weaker coupled to electrodes that the HOMO-1, and this is why the p-type conduction of the CnA junction is dominated by the HOMO-1 and not by the HOMO

$$\begin{aligned} I &= G_0 \frac{\Gamma^2_{HOMO}(V)}{\left[E^{emb}_{HOMO}(V) - E_F\right]^2 - (eV/2)^2} V \qquad \text{(S3)} \\ &+ G_0 \frac{\Gamma^2_{HOMO-1}\cancel{(V)}}{\left[E^{emb}_{HOMO-1}(V) - E_F\right]^2 - (eV/2)^2} V \\ &\xlongequal{\Gamma_{HOMO}(V) \ll \Gamma_{HOMO-1}} \frac{G_0 V \Gamma^2_{HOMO-1}}{\underbrace{\left[E_{HOMO-1}(V) - E_F\right]^2}_{\varepsilon_0^{trans}} - (eV/2)^2} \qquad \text{(S4)} \end{aligned}$$

To sum up, eq (S4) (which is just eq (1)) of the main text in specific notations) does apply and not eq (S1).

Table S2: HOMO energy offset deduced from transport ($\varepsilon_h^{\text{tran}}$, eV) and UPS ($\varepsilon_h^{\text{UPS}}$, eV) for CnT CF-AFM junctions reproduced form ref 44

electrode	quantity	CnT			
	n	8	9	10	12
Ag/Ag	$\varepsilon_h^{\text{trans}} = \lvert\varepsilon_0^{trans}\rvert$	1.13	1.14	1.13	1.14
	$\varepsilon_h^{\text{UPS}} = \lvert\varepsilon_0^{UPS}\rvert$	1.29	1.27	1.30	1.30
Au/Au	$\varepsilon_h^{\text{trans}} = \lvert\varepsilon_0^{trans}\rvert$	1.01	0.94	0.97	0.98
	$\varepsilon_h^{\text{UPS}} = \lvert\varepsilon_0^{UPS}\rvert$	0.92	0.91	0.90	0.92
Pt/Pt	$\varepsilon_h^{\text{trans}} = \lvert\varepsilon_0^{trans}\rvert$	0.91	0.88	0.83	0.87
	$\varepsilon_h^{\text{UPS}} = \lvert\varepsilon_0^{UPS}\rvert$	0.85	0.89	0.83	0.87

HOMO and HOMO-1 energies of isolated CnA molecules: comparison between OVGF and other methods

The small differences between the various MO energy offsets entering the analysis of the present paper makes it clear why utilizing a very accurate quantum chemical method is of paramount importance. The method based on the OVGF is a very elaborate many-body methods wherein the one-electron Green function includes expansions of the self-energy entering the electronic Dyson equation.[94] This expansion is exact up to third order in the Coulomb electronic repulsion. Moreover, this third order expansion is supplemented by a geometrical approximation to higher (than third) orders in the electronic repulsion. Table S3 and Figure S8 give a flavor on how large are the differences between the OVGF estimates of the HOMO and HOMO-1 energies and other methods. Letting alone enormous differences from DFT values based on the Kohn-Sham (KS) orbital energies (amounting up to a few eV), we note that the $\Delta-$DFT difference method [110] (a method often considered “acceptable”) give an estimate for the HOMO (HOMO-1 cannot be computed using this method) that deviates from the OVGF value by about 0.7 eV. Even popular Møller-Plesset (MP) methods yield values differing by $\sim 0.1 - 0.2\,$eV.

Because what really matters in the analysis presented in the main text is the difference between the HOMO and HOMO-1 energies, with one exception (label “OVGF(zpm)”, second numerical line in Table S3), the theoretical values presented in Table S3 do not contain corrections due to zero-point motion (zpm). The OVGF-based HOMO energies including zpm corrections shown in the second numerical line in Table S3) make comparison with the experimental ionization potentials (IP) [116] (first numerical line in Table S3). As visible in Table S3, the theoretical results perfectly reproduce the experimental data.

Table S3: Values in eV of HOMO and HOMO-1 energies (E^0_{HOMO} and E^0_{HOMO-1}) for isolated CnA molecules calculated at various levels of theory (see ref. 110) indicated in the table. Notice that, within the experimental errors, the OVGF values including corrections due to zero-point motion (zpm) perfectly reproduce the experimental data for the lowest ionization potential (IP) *via* photoionization mass spectroscopy: Rang, S.; Martinson, E., "Ionization potentials of unsaturated hydrocarbons. 3. n-Alkyunes C8-C12.", Eesti. NSV Tead. Akad. Toim. Keem., 1978, 44 cited after ref. 116

Property	Method	C8A	C9A	C10A	C12A
$-$IP= E_{HOMO}	Experiment	-9.91 ± 0.02	-9.90 ± 0.02	-9.90 ± 0.02	-9.89 ± 0.02
HOMO	OVGF(zpm)	9.928	-9.895	-9.891	-9.883
HOMO	OVGF	-9.988	-9.984	-9.981	-9.977
HOMO-1	OVGF	-10.086	-10.085	-10.084	-10.083
δ	OVGF	0.098	0.101	0.103	0.106
HOMO	HF-Koopman	-10.351	-10.349	-10.348	-10.347
HOMO-1	HF-Koopman	-10.391	-10.391	-10.390	-10.389
δ	HF-Koopman	0.040	0.042	0.042	0.042
HOMO	Δ-HF	-8.795	8.793	-8.791	-8.789
HOMO	Koopman-B3LYP	-7.402	-7.400	-7.399	-7.397
HOMO-1	Koopman-B3LYP	-7.420	-7.419	-7.418	-7.418
δ	Koopman-B3LYP	0.017	0.018	0.020	0.020
HOMO	Δ-B3LYP	-9.384	-9.296	-9.216	-9.080
HOMO	EOM-MP2	-10.008			
HOMO-1	EOM-MP2	-10.174			
δ	EOM-MP2	0.167			
HOMO	Δ-MP2	-10.345	-10.343	-10.341	-10.339
HOMO	Δ-MP3	-10.024	-10.221		-10.019
HOMO	Δ-MP4D	-10.080	-10.078		-10.075
HOMO	Δ-MP4DQ	-10.041	-10.039		-10.036
HOMO	Δ-MP4SDQ	-10.060	-10.058		-10.054

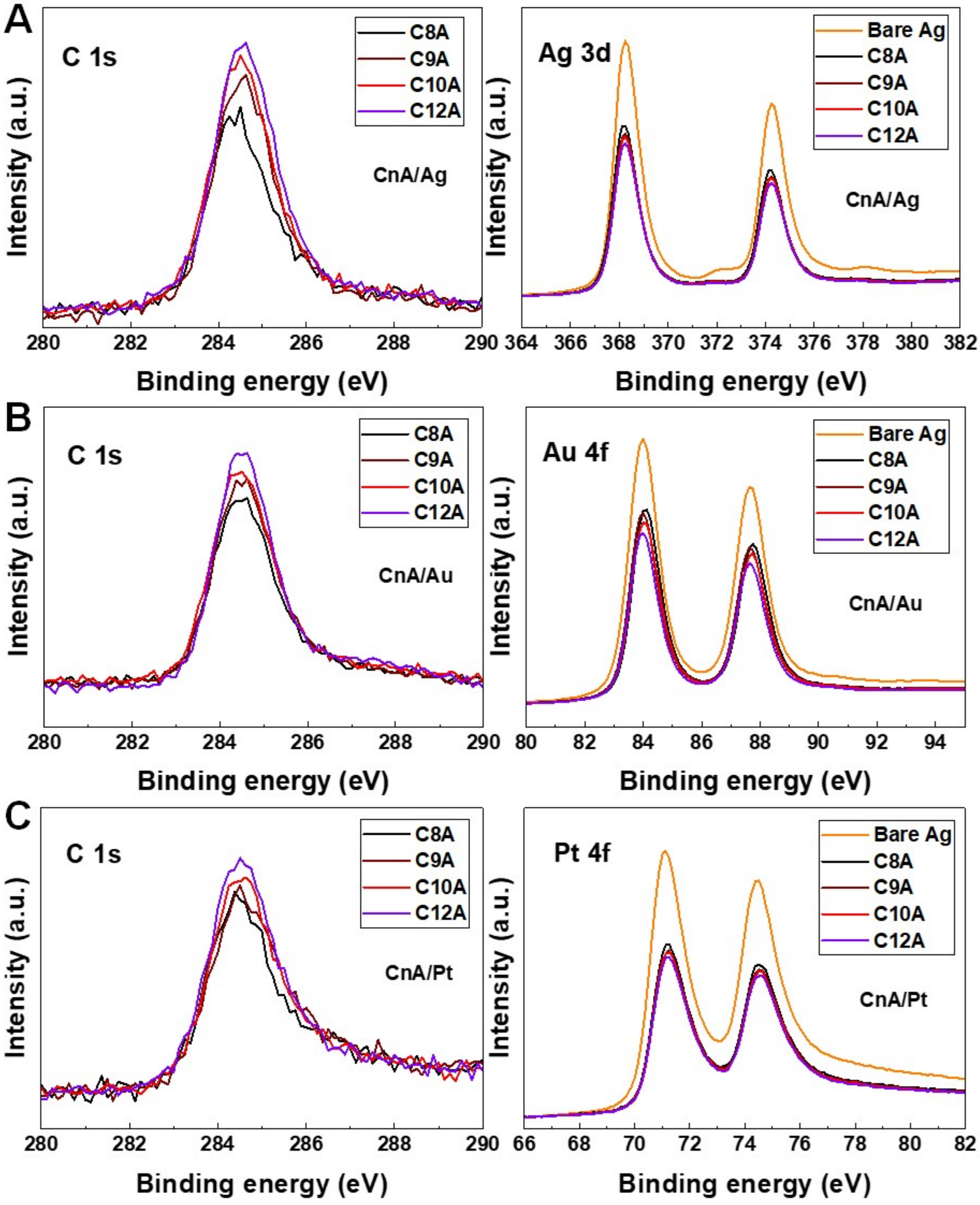

Figure S4: XPS spectra of (A) CnA /Ag, (B) CnA /Au, (C) CnA /Pt (n=8, 9, 10, 12).

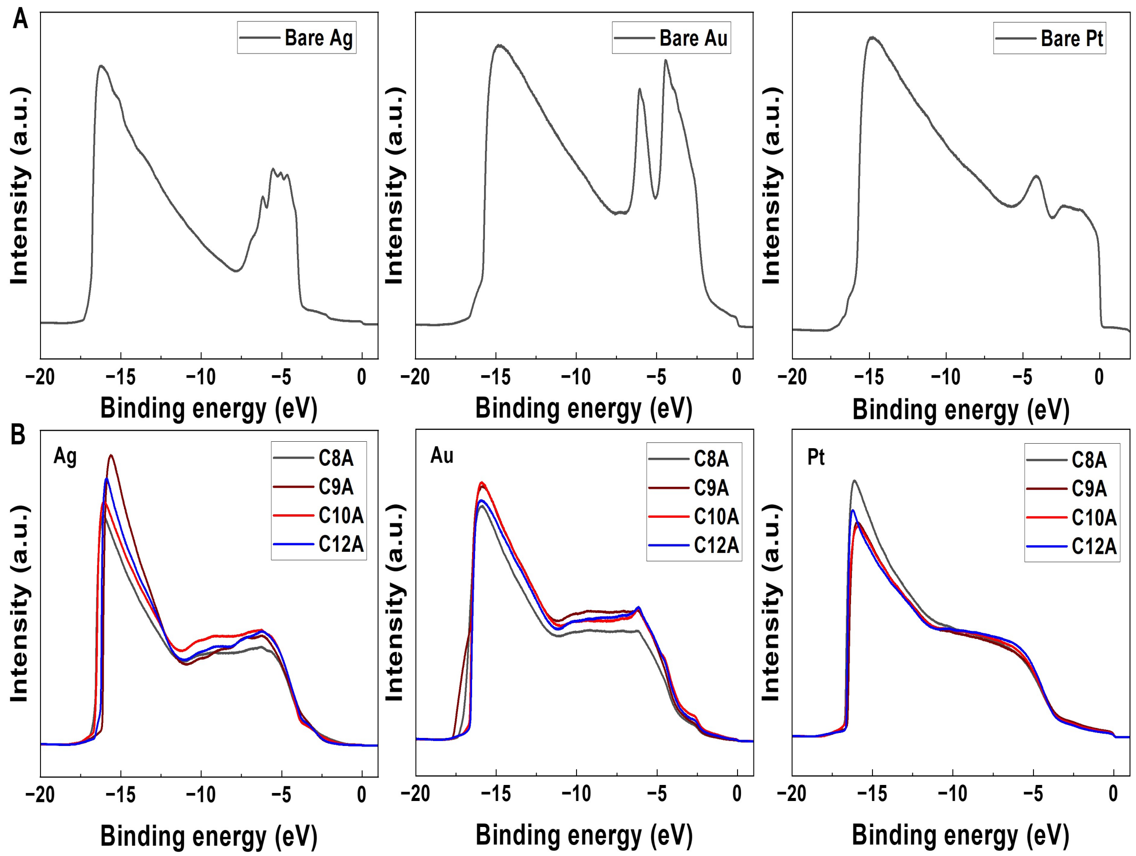

Figure S5: UPS spectra of (A) bare Ag, Au and Pt, (B) CnA on Ag, Au and Pt.

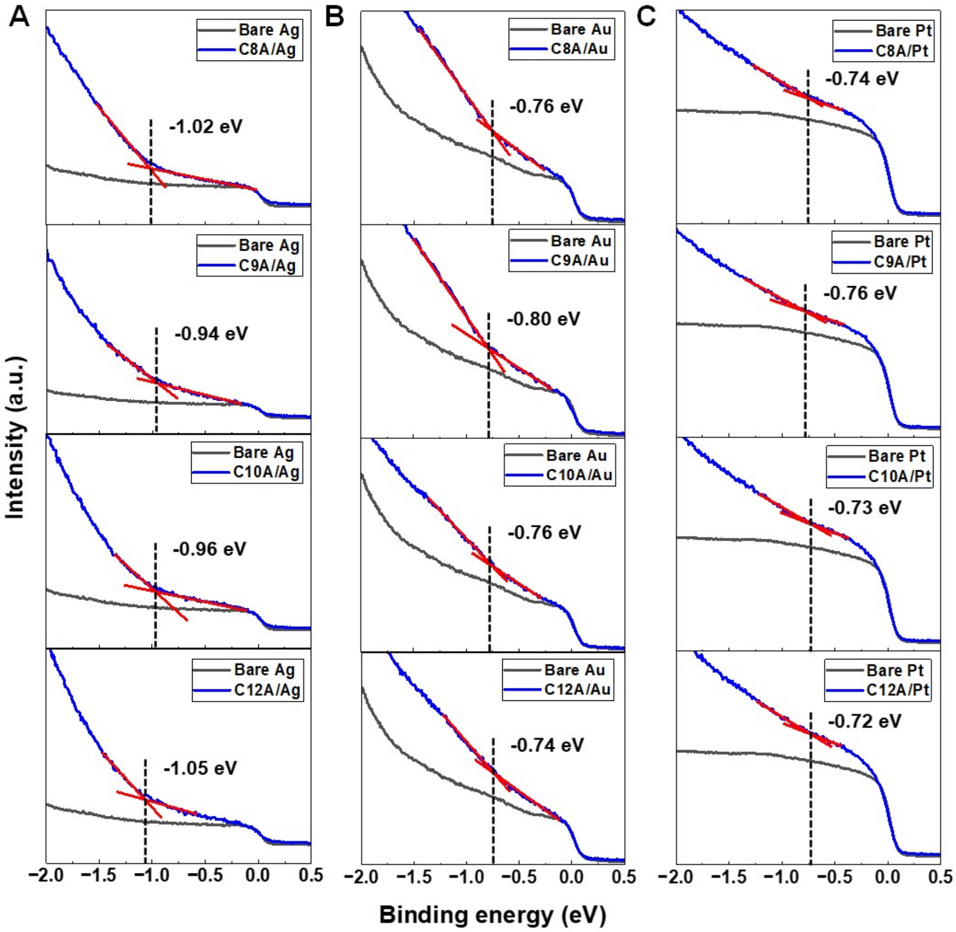

Figure S6: UPS spectra of CnA on (A) Ag, (B) Au, and (C) Pt substrates (n= 8, 9, 10, 12). Binding energies are referenced to the Fermi level, $E_F = 0$,eV. The spectral intensity of SAM-coated metal substrates was normalized to the intensity of bare metal substrates at 0 eV. The crossing points of the red lines indicate the onsets of the HOMOs; the values in eV represent the HOMO-Fermi energy offsets $|\varepsilon_0^{\text{CnA SAM}}|$.

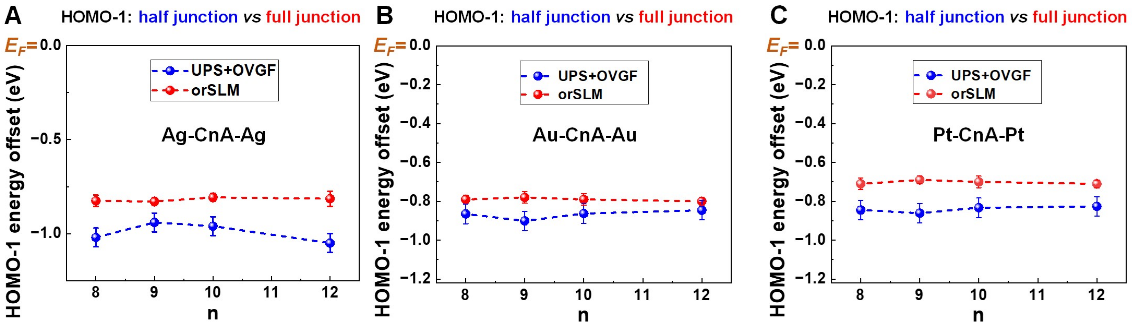

Figure S7: The HOMO-1 offset directly extracted from transport data clearly differs from the HOMO-1 offset deduced by combining UPS data and OVGF quantum chemical calculations.[95,96] This demonstrates that, contrary to alkane [44] and oligophenylene [43] mono- and di-thiols, the ("second") tip electrode brings about an additional nonnegligible (measurable) HOMO shift towards the metal Fermi level. Our data by no means indicate an energy difference monotonically decreasing with the molecular size, a fact which rules out any image charge effects.

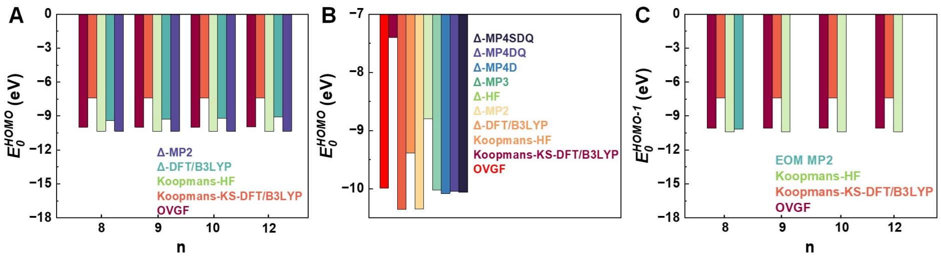

Figure S8: (A) Values of the HOMO energy E^0_{HOMO} for isolated CnA molecules of computed via OVGF depicted along with estimates using the most popular methods utilized in the literature indicated in the legends for the molecular sizes n considered in this paper. (B) Additional values of E^0_{HOMO} for 1-decyne (C10A). (C) Values of the HOMO-1 energy E^0_{HOMO-1} for isolated CnA molecules of computed via OVGF and a few other methods enabling estimation of this quantity.
